# Immune response to hepatitis B vaccine among children under 5 years in Africa: a meta-analysis

**DOI:** 10.1186/s41182-024-00594-4

**Published:** 2024-04-01

**Authors:** Babayemi O. Olakunde, Ijeoma M. Ifeorah, Daniel A. Adeyinka, Olubunmi A. Olakunde, Temitayo Ogundipe, John O. Olawepo, Echezona E. Ezeanolue

**Affiliations:** 1 Department of Community Prevention and Care Services, National Agency for the Control of AIDS, Abuja, Nigeria; 2 Center for Translation and Implementation Research, University of Nigeria Nsukka, Enugu, Nigeria; 3 Present Address: Department of Population and Community Health, School of Public Health, University of North Texas Health Science Center, 3500 Camp Bowie Blvd., Fort Worth, TX 76107 USA; 4 Department of Medical Laboratory Sciences, University of Nigeria Nsukka, Enugu, Nigeria; 5 Department of Research, Saskatchewan Health Authority, Saskatoon, SK Canada; 6 Department of Public Health, Federal Ministry of Health, Abuja, Nigeria; 7 Department of Disease Control and Immunization, Ondo State Primary Health Care Development Agency, Ondo, Nigeria; 8 Sentara Williamsburg Regional Medical Center, Williamsburg, VA USA; 9 Department of Health Sciences, Northeastern University, Boston, MA USA; 10 Healthy Sunrise Foundation, Nevada, USA

## Abstract

**Background:**

Hepatitis B virus (HBV) infection in Africa is mostly acquired before the age of 5 years through vertical or horizontal routes. While all the countries in the World Health Organization African region have introduced HBV vaccination into their national immunization programs, the rate of protective immune response to HBV vaccine among children in Africa has not been systematically synthesized. In this study, we estimated the HBV vaccine seroprotection rate (defined as anti-HBs titer ≥ 10 IU/L) and the associated factors among under-five children who completed a primary series of HBV vaccination in Africa.

**Methods:**

We systematically searched PubMed, Web Science, and Scopus databases from inception to May 2022 for potentially eligible studies. The pooled seroprotection rate was estimated using a random-effects model with Freeman–Tukey double arcsine transformation and the associated factors were examined using odds ratio estimated by the DerSimonian and Laird method.

**Results:**

From the 1063 records identified, 29 studies with a total sample size of 9167 under-five children were included in the meta-analysis. The pooled seroprotection rate was 89.23% (95% CI   85.68–92.33%, *I*^2^ = 95.96%, *p* < 0.001). In the subgroup analyses, there was a significant difference in the rate by the assay method, vaccine dose, and vaccine combination. HIV-positive children had lower odds of achieving seroprotection when compared with HIV-negative children (OR = 0.22, 95%CI   0.12–0.40).

**Conclusions:**

The majority of under-five children in Africa achieved seroprotection after completing three or four doses of HBV vaccine. However, the rate was lower among children living with HIV. This calls for interventions to timely identify and address nonresponse to HBV vaccine, particularly among immunosuppressed children.

**Supplementary Information:**

The online version contains supplementary material available at 10.1186/s41182-024-00594-4.

## Main text

### Background

Despite the decline in the burden of hepatitis B virus (HBV) infection, it remains a major global public health problem [1, 2]. Globally, there were approximately 1.5 million new HBV infections and 820,000 HBV-related deaths in 2019 [3]. Africa continues to be disproportionately affected by HBV infection, accounting for about 67% of the new infections in 2019 [3]. HBV infection in Africa is mostly acquired before the age of 5 years through vertical or horizontal routes [4–7]. Compared with HBV infection acquired in adulthood which leads to chronic hepatitis in less than 5% of cases, about 80–90% of persons infected in the first year of life and 30% of those infected before the age of 6 years develop chronic hepatitis [8]. Thus reducing new infections among children is one of the global targets of the ongoing efforts aimed at eliminating HBV infections as a major public health threat in Africa [9].

The availability of safe and effective HBV vaccines [10–13] has been pivotal to the prevention and control of HBV infection, globally [14–16]. HBV vaccine, which can be plasma-derived (although no longer in use) or recombinant DNA-derived, is available as a monovalent vaccine or in combination with other vaccines [17, 18]. Given the benefits, the World Health Organization (WHO) recommends a hepatitis B vaccine birth dose (HepB-BD) for newborns, followed by two or three doses given at least four weeks apart to complete the primary series [4, 18]. HBV vaccination is also recommended for persons at increased risk such as HIV-infected persons, men who have sex with men, healthcare workers, persons with multiple sexual partners, injecting drug users, and persons who frequently require blood or blood products [18]. As recommended, all 47 countries in the WHO African region have introduced HBV vaccination into their national immunization programs [19], with the coverage of 3 doses of HBV vaccine estimated at 72% in 2022 [20]. However, as of 2022, only 15 (32%) of the African countries had introduced HepB-BD vaccine into their national immunization programs [21], with an estimated coverage of 18% in the region [20].

Notwithstanding the efficacy of the HBV vaccine [10–13], the ability to mount a protective immune response (defined as a hepatitis B surface antibody [anti-HBs] titer ≥ 10 IU/L) [22] following vaccination varies among vaccinees. Approximately 5–10% of healthy persons (referred to as non-responders) do not develop seroprotective anti-HBs level after completing HBV vaccine primary series [23, 24]. Studies have shown that factors such as age, sex, body mass index, vaccination schedule, site, dose, route of administration, and the brand of vaccine affect the immune response to HBV vaccine [24–31]. Evidence also indicates that the immune response to HBV vaccine is lower in individuals with immunosuppressive or chronic diseases, such as HIV, chronic renal failure, diabetes, celiac disease, or chronic liver disease [25, 32–35]. For those at high risk of HBV infection, post-vaccination serologic testing (PVST) within one to two months after the final dose of vaccine is recommended to identify non-responders and evaluate the need for revaccination [18]. Strategies to improve immune response among non-responders include increased vaccination dose, additional vaccination cycles, an alternative route of administration, or the use of adjuvants [35–38].

An increasing number of studies have reported on the immune response to HBV vaccine among children in Africa; however, the proportion that achieves seroprotective level has not been systematically synthesized. Previous meta-analytic studies on the immune response to HBV vaccine [24–28, 32–34, 39–42] did not focus on children or include studies from Africa, limiting the evidence on this vulnerable population who are at increased risk of developing chronic HBV infection. In a literature review of recombinant HBV vaccine among infants, the immune response rate ranged from 50% to 100% with a median of 98%. However, the review only considered trials, monovalent vaccines, and infants in the first 30 days of life [43]. Insight into the immune response to HBV vaccine among African children can inform strategies to improve its delivery or monitoring for effectiveness and maximum impact in the high-burden continent.

The objective of this review was to estimate the seroprotection rate (i.e., anti-HBs titer ≥ 10 IU/L) and the associated sociodemographic and clinical factors among under-five children who completed a primary series of HBV vaccination in Africa.

## Methods

### Design

This systematic review and meta-analysis was performed and reported using the guidelines for Preferred Reporting Items for Systematic Reviews and Meta-Analyses (PRISMA) [44]. This review was registered in the International Prospective Register of Systematic Reviews (PROSPERO) (registration no: CRD42022299988).

### Information sources and search strategy

We systematically searched PubMed, Web Science, and Scopus databases from inception to May 2022 for potentially eligible articles using search terms relating to hepatitis B; vaccination; immunogenicity; children; and African countries (see Additional file 1). No language restriction was applied. We also searched the bibliographies of the identified articles for other potentially eligible articles.

### Inclusion and exclusion criteria

Articles were eligible for inclusion if: (i) they included under-five children; (ii) the participants completed a three-dose schedule of HBV vaccine primary series or birth dose vaccine plus two or three doses; (iii) they reported anti-HBs titer following the last dose of the vaccine series; and (iv) the study design was experimental or observational. We excluded studies that: (i) were not conducted in Africa, (ii) the anti-HBs were not quantified or the cutoff of ≥ 10UI/L was not reported; (iii) used the same data (we retained the one with more information regarding the inclusion criteria); or (iv) the participants did not receive HBV-specific vaccine, did not complete the HBV vaccine series, or received HBV vaccine booster dose after the completion of primary series. We also excluded conference abstracts and studies where the full articles or anti-HBs level data from figures could not be retrieved.

### Study selection and abstraction

Two authors (BOO and OAO) first independently screened the title and abstracts of the articles and the full articles of those deemed eligible were retrieved and screened for inclusion. Articles were only retrieved and included if there was an agreement between the two authors. Disagreements between the two authors were resolved by a third author (DAA). Three authors (BOO, TO, and OAO) extracted data from the articles using a pretested tool that included Information such as the first author’s surname, publication year, study location, study design, study population, number of participants, participants' age, vaccine schedule, vaccine dose, vaccine type, vaccine combination, and assay method. The number of participants that had anti-HBs ≥ 10 IU/L was extracted or calculated from the included studies. Disaggregated anti-HBs ≥ 10 IU/L by sociodemographic and clinical factors were also abstracted. We grouped the study locations into regions (North; Central and West; and East and Southern Africa). In the description of the study population, we classified participants according to their reported health conditions or disease exposure. We classified participants as “healthy” if they were not primarily recruited based on or described by any specific health condition or exposure to a disease. For studies that assayed anti-HBs at multiple timepoints, we used the first timepoint.

### Quality assessment

The quality of the articles included in the study was assessed using the Effective Public Health Practice Project (EPHPP) Quality Assessment tool [45]. The tool evaluates and rates the quality of quantitative studies under the following categories: study design, analysis, withdrawals and dropouts, data collection practices, selection bias, invention integrity, blinding as part of a controlled trial, and confounders. The articles were rated “strong,” “moderate,” and “weak” per the EPHPP guide for component and global rating. However, it was decided a priori not to exclude any study based on the quality rating.

### Analysis

The meta-analysis for the pooled seroprotection rate was conducted using the procedure for binomial data [46]. Due to the statistical heterogeneity, the pooled rate was estimated using a random-effects meta-analysis model with Freeman–Tukey double arcsine transformation [47]. Sociodemographic and clinical factors reported by at least two articles were included in the meta-analysis and their associations with seroprotection were examined using odds ratio (OR) estimated by the DerSimonian and Laird method [48]. Statistical heterogeneity between the studies was assessed using Cochran’s *Q* statistic, with a *p* value < 0.1 set as the level of statistical significance [49]. *I*^2^ was also used to quantify the heterogeneity. We considered *I*^2^ statistic values of 50% or more as substantial heterogeneity [49]. Subgroup analyses were performed for group comparisons. The studies were grouped by region, assay method, vaccine dose, vaccine type, and vaccine combination. Meta-regression was performed to assess the effect of continuous characteristics (study sample size and publication year) on the seroprotection rate [50]. It was also used to assess the proportion of between-study variance explained by vaccine dose, vaccine type, assay method, region, vaccine combination, sample size, and publication year. Leave-one-out meta-analysis was used to investigate the influence of each study on the overall effect-size estimate and to identify influential studies. Publication bias was assessed using a funnel plot and Egger’s test [51]. The meta-analysis was conducted using STATA V.17.0 for Windows (Stata Statistical Software: Release 17. College Station, TX: StataCorp).

### Search results

**Fig. 1:**
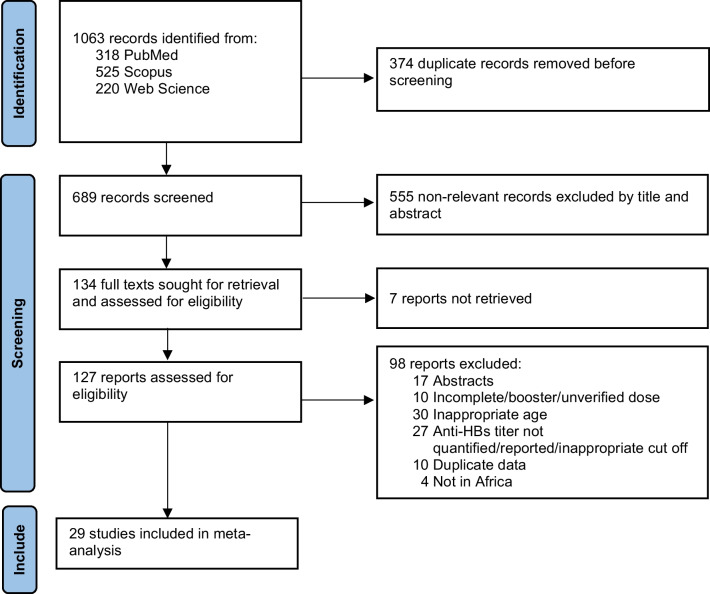
PRISMA flow diagram of the process of study identification and selection

The PRISMA flow diagram for the study selection is shown in Table 1. A total of 1063 records were identified through three databases. Following the removal of 374 duplicates, the titles and abstracts of 689 records were screened and 555 were deemed ineligible. The full−text articles of 134 reports were retrieved and assessed for eligibility. Twenty−nine reports were included in the study and 98 articles were excluded with reasons illustrated in Fig. 1.

**Table 1:** Summary characteristics of studies included in the review

First Author, year of publication	Study design	Country	Region	Primary population	Age of participants (under five)	Vaccine schedule	Vaccine doses	Vaccine combination	Vaccine type	Assay method	Sample size (under five)	Quality assessment
Abushady, 2011 [52]	Cross-sectional	Egypt	NA	Healthy	2–3 years	2, 4, and 6 months	3 doses	Monovalent	Recombinant	Enzyme immunoassay	200	Weak
Accrombess, 2020 [53]	Cross-sectional	Benin	WCA	Healthy*	9 months	6, 10, and 14; 0, 6, 10, and 14 weeks	3 and 4 doses	Pentavalent; Monovalent	Recombinant	Enzyme immunoassay	136	Moderate
Anutebeh, 2021 [54]	Cross-sectional	Cameroun	WCA	Healthy**	6–9 months	6, 10, and 14 weeks; 0, 6, 10, and 14 weeks	3 and 4 doses	NR	NR	Enzyme immunoassay	161	Weak
Apiung, 2017 [55]	Cross-sectional	Ghana	WCA	Healthy	5–32 months	6, 10, and 14 weeks	3 doses	Pentavalent	Recombinant	Enzyme immunoassay	424	Moderate
Aspinall, 1998 [56]	Longitudinal	South Africa	ESA	Healthy	Infants	6, 10, and 14 weeks	3 doses	Monovalent	Plasma-derived	Enzyme immunoassay	186	Weak
Aspinall, 2012 [57]	RCT (phase III)	South Africa	ESA	Healthy	42–64 days	6, 10, and 14 weeks	3 doses	Pentavalent	Recombinant	Enzyme immunoassay	320	Strong
Baroncelli, 2021 [58]	Longitudinal	Malawi	ESA	HIV-exposed***	Infants	6, 10, and 14 weeks	3 doses	Pentavalent	NR	Enzyme immunoassay	111	Moderate
Coursaget, 1992 [59]	Longitudinal	Senegal	WCA	Healthy	Infants	2, 4, and 9 or 10 months	3 doses	Monovalent	Plasma-derived; Recombinant	Radioimmunoassay	122	Weak
El-Asheer, 2015 [60]	Cross-sectional	Egypt	NA	Healthy and acutely/chronically ill	≤ 1 year	2, 4, and 6 months	3 doses	NR	Recombinant	Enzyme immunoassay	29	Weak
Fortuin, 1993 [61]	Cross-sectional	Gambia	WCA	Healthy	3–4 years	0, 2, 4 and 9 months	4 doses	NR	Plasma-derived	Radioimmunoassay	24	Weak
Hodgson, 2008 [62]	RCT (phase II)	Ghana	WCA	Healthy	6–8 weeks	6, 10, and 14 weeks	3 doses	Pentavalent, Heptavalent	Recombinant	Enzyme immunoassay	244	Strong
Koen, 2021 [63]	RCT (open-label, phase III)	South Africa	ESA	HIV-exposed infected and HIV-exposed uninfected	5–8 weeks	6, 10, and 14 weeks	3 doses	Hexavalent	Recombinant	Chemiluminescence immunoassay	63	Strong
Madhi, 2011a [64]	RCT (open-label, phase III)	South Africa	ESA	Healthy	> 24 h	6, 10, and 14 weeks	3 doses	Monovalent	Recombinant	Chemiluminescence immunoassay	196	Moderate
Madhi, 2011b [65]	RCT (open-label, phase III)	South Africa	ESA	Healthy	0–3 days	0, 6, 10, and 14 weeks; 6, 10, and 14 weeks	3 and 4 doses	Hexavalent and monovalent	Recombinant	Chemiluminescence immunoassay	555	Moderate
Magoni, 2009 [66]	Cross-sectional	Ivory Coast	WCA	Healthy	12–59 months	6, 10, and 14 weeks	3 doses	NR	NR	Enzyme immunoassay	609	Weak
Mancinelli, 2018 [67]	Longitudinal	Malawi	ESA	HIV-exposed****	0 week	6, 10, and 14 weeks	3 doses	NR	NR	Enzyme immunoassay	58	Weak
Mbuthia, 2018 [68]	Cross-sectional	Kenya	ESA	General and HIV-infected	< 24–48 months	6, 10, and 14 weeks	3 doses	Pentavalent	Recombinant	Enzyme immunoassay	217	Weak
Metodi, 2010 [69]	Cross-sectional	Tanzania	ESA	General*****	2–59 months	4, 8, and 12 weeks	3 doses	Tetravalent	Recombinant	Enzyme immunoassay	279	Weak
Newton, 2007 [70]	RCT (open-label)	Ghana	WCA	Healthy	6–18 weeks	6, 10, and 14 weeks	3 doses	Pentavalent	Recombinant	Enzyme immunoassay	888	Strong
Nlend, 2016 [71]	Cross-sectional	Cameroun	WCA	Healthy, HIV-exposed uninfected, HIV infected	6–59 months	A series of three-monthly doses	3 doses	Pentavalent	Recombinant	Enzyme immunoassay	82	Weak
Ouedraogo, 2013 [72]	Cross-sectional	Burkina Faso	WCA	Healthy	6–18 months	A series of three-monthly doses	3 doses	Pentavalent	Recombinant	Immunofluorescence assay	200	Weak
Rey-Cuille, 2012 [73]	Cross-sectional	Cameroun and Senegal	WCA	Acutely/chronically ill	< 4 years	6, 10, and 14 weeks	3 doses	Pentavalent	Recombinant	Enzyme immunoassay	242	Moderate
Salama, 2015 [74]	Cross-sectional	Egypt	NA	Healthy	< 5 years	2, 4 and 6 months	3 doses	NR	Recombinant	Enzyme immunoassay	1114	Moderate
Schoub, 2002 [75]	Cross-sectional	South Africa	ESA	Healthy	18 months	6, 10, and 14 weeks	3 doses	Monovalent	Plasma	Enzyme immunoassay	769	Moderate
Shindano, 2019 [76]	Cross-sectional	Democratic Republic of the Congo	WCA	Healthy	6–12 months	6, 10, and 14 weeks	3 doses	Pentavalent	Recombinant	Enzyme Immunoassay	200	Weak
Simani, 2014 [77]	Cross-sectional (archived serum samples)	South Africa	ESA	Healthy, HIV-exposed infected, and HIV-exposed uninfected	6–12 weeks	6, 10, and 14 weeks	3 doses	Monovalent	Recombinant	Immunofluorescence assay	482	Moderate
Tsebe, 2001 [78]	Cross-sectional	South Africa	ESA	Healthy	8–48 months	6, 10, and 14 weeks	3 doses	Monovalent	Plasma-derived	Enzyme immunoassay	569	Weak
Valéa, 2018 [79]	RCT (open-label phase III)	Burkina Faso and Ghana	WCA	Healthy	8–12 week	8, 12 and 16 weeks	3 doses	Monovalent	Recombinant	Chemiluminescence immunoassay	253	Strong
Whittle, 1991 [80]	Cross-sectional	Gambia	WCA	Healthy	0–4 years	2, 4, and 6 months; 1, 2, 4, and 9 months	3 and 4 doses	NR	Recombinant	Radioimmunoassay	434	Weak

*RCT*  Randomized Controlled Trial; *NA*  North Africa; *ESA*  East and Southern Africa; *WCA*  West and Central Africa; *HBV*  Hepatitis B virus; *HIV*  Human immunodeficiency virus; *EBV*  Epstein–Barr virus; *NR*  Not reported

*7.1% of the study population HBV-exposed; **3.7% HIV-exposed; ***88.7% EBV-infected; ****8.3% HBV-exposed; *****10.5% HIV-infected; ****** 2.0% HIV-exposed and 3.0% HBV-exposed

### Characteristics of the included studies

**Fig. 2:**
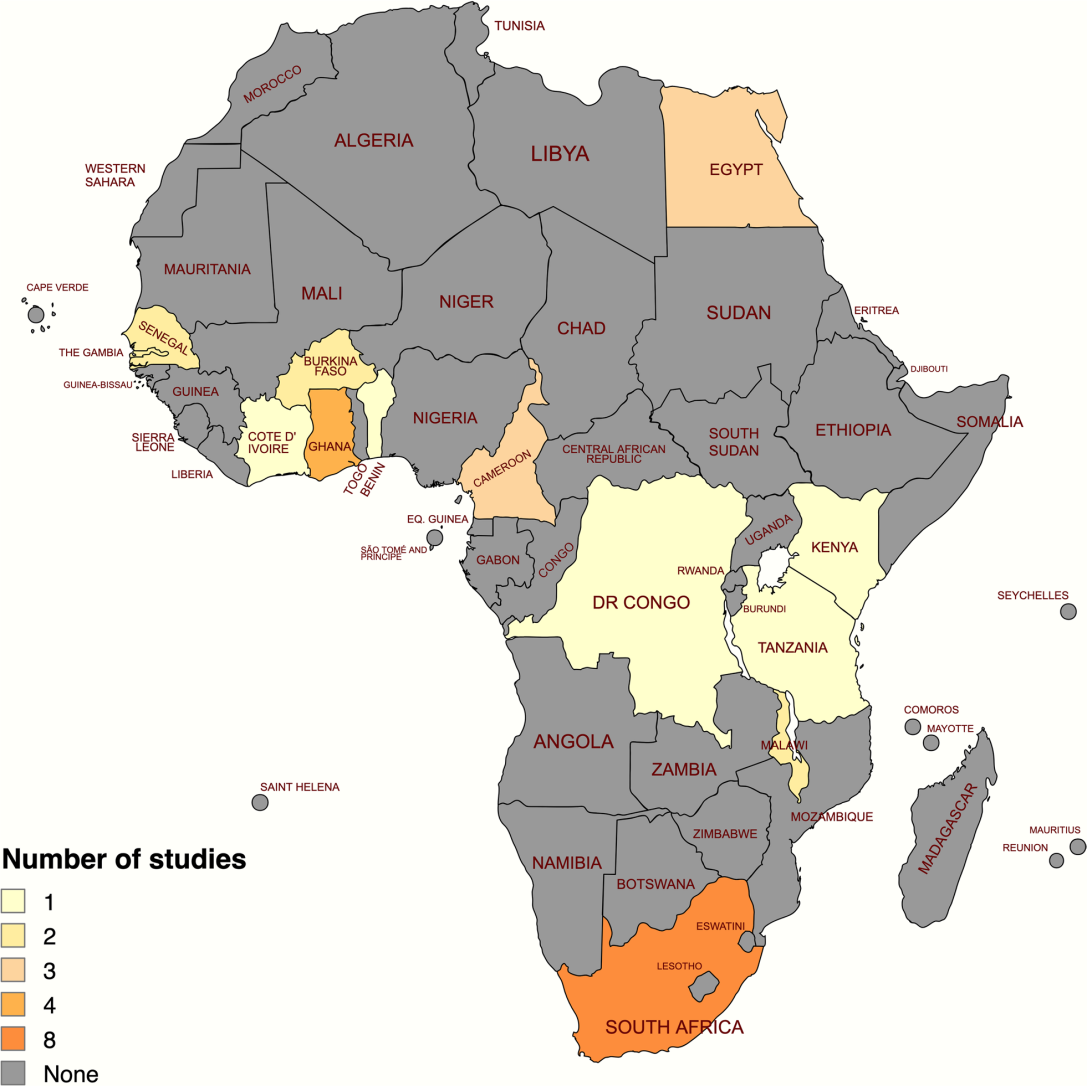
Distribution of the included studies

All the articles were published in English. The publication year of the studies ranged from 1991 to 2021 as shown in Table 1. The studies included had a total of 9167 under-five children. Eighteen of the 29 studies were cross-sectional studies, four studies were longitudinal, and seven studies were clinical trials (see Table 1). The studies were conducted in the following countries: Benin (*n*=1), Burkina Faso (*n*=1), Cameroun (*n*=2), Democratic Republic of Congo (*n*=1), Egypt (*n*=3), Gambia (*n*=2), Ghana (*n*=3), Ivory Coast or Côte d'Ivoire (*n*=1), Kenya (*n*=1), Malawi (*n*=2), Senegal (*n*=1), South Africa (*n*=8), Tanzania (*n*=1), Burkina Faso and Ghana (*n*=1), and Cameroun and Senegal (*n*=1) (see Fig. 2). The population in the studies included healthy, HIV−exposed, HIV−infected, HIV−exposed uninfected, and acutely/chronically ill children. In 24 studies, the participants completed 3 doses, whereas in 1 study it was 4 doses of HBV vaccine. In 4 studies, the participants completed either 3 or 4 doses of HBV vaccine. The types of combination vaccines reported in the included studies were: monovalent (*n*=8), pentavalent (*n*=9), pentavalent and monovalent (*n*=1), hexavalent and monovalent (*n*=1), hexavalent (*n*=1), tetravalent (*n*=1), and pentavalent and heptavalent (*n*=1). The vaccine combination was not reported in 7 studies. Recombinant vaccine was used in 19 studies, plasma−derived vaccine in 4 studies, both recombinant and plasma−derived vaccine in 1 study. Five studies did not report the vaccine type. The vaccine schedule varied across the studies, with the 6, 10, and 14−week schedule mostly reported in the studies (*n*=19). A total of 20 studies used enzyme immunoassays for the quantification of the antibody response to HBV vaccine. Most of the studies were rated weak (*n*=15), largely because of their cross−sectional nature.

### Seroprotection rate

**Fig. 3:**
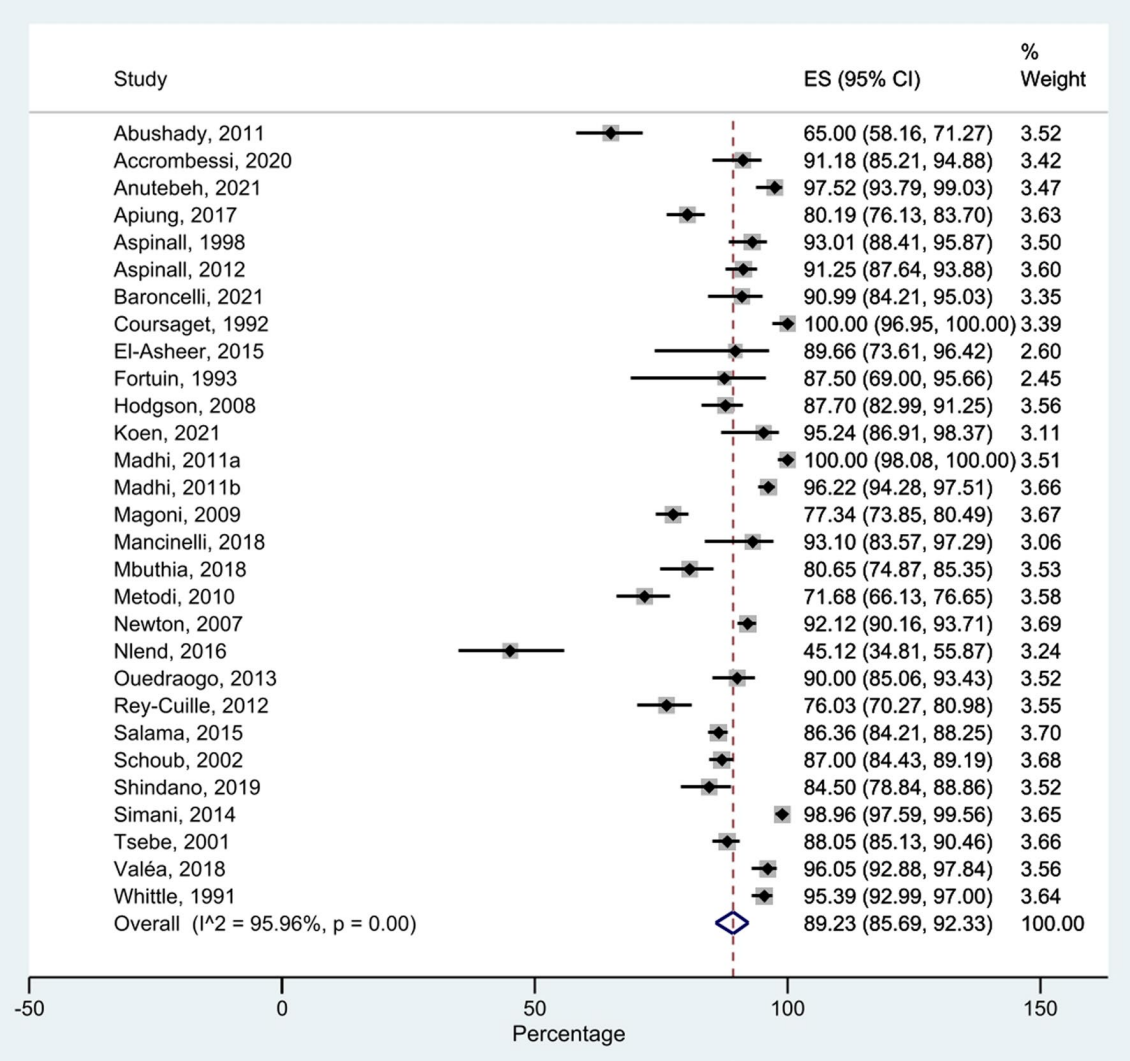
Forest plot of the seroprotection rates after HBV vaccination among children under 5 years in Africa

The seroprotection rates after HBV vaccination in the included studies ranged from 45% to 100% (Fig. 3). The pooled rate was 89.23% (95% confidence interval [CI] 85.69–92.33%). The homogeneity test indicated the presence of heterogeneity in the data (*I*2 = 95.96%, *p* <0.001).

### Subgroup analysis

Table 2 (Additional file 2: Fig. S1) shows the subgroup analysis of the seroprotection rate by region, assay method, vaccine dose, vaccine type, and vaccine combination. The subgroup differences by region and vaccine type were not statistically significantly different. However, there was a significant difference in the assay method, ranging from 97.61% (95%CI   94.30–99.60%) in studies that used chemiluminescence assay to 85.07% (95%CI   81.20–88.58%) in studies that used enzyme immunoassay. The subgroup analysis showed a significant difference between three vaccine doses (89.00% [95%CI   85.37–92.17%]) and four vaccine doses (97.17% [95%CI   93.29–99.62%]). There was also a significant subgroup difference by vaccine combination, ranging from 95.55% (95%CI   92.71–97.76%) in studies that used hexavalent vaccine to 71.68% (95%CI   66.13–76.65%) in one study that used tetravalent vaccine.

**Table 2:** Subgroup analysis of the seroprotection rate after HBV vaccination among children under 5 years in Africa

Subgroups	Number of studies	Sample size	Pooled rate (95% CI)	*I*^2^ (*p* value)	*p* value (subgroup differences)
*Region*					
North	3	1343	80.69% (62.87–93.81%)	NC	0.232
West and Central	14	4019	88.15% (82.49–92.85%)	95.86 (< 0.01)	
East and Southern	12	3805	92.01% (86.95–95.95%)	95.85 (< 0.01)	
*Assay method*					
Enzyme immunoassay	19	6229	85.07% (81.20–88.58%)	93.53 (< 0.01)	< 0.001
Radioimmunoassay	4	1189	92.73% (77.69–80.49%)	97.66 (< 0.01)	
Chemiluminescence assay	4	1067	97.61% (94.30–99.60%)	83.53 (< 0.01)	
Immunofluorescence assay	2	682	97.27% (95.88–98.40%)	NC	
*Vaccine dose*					
Three doses	28	8586	89.00% (85.37–92.17%)	95.84 (< 0.01)	0.005
Four doses	5	464	97.17% (93.29–99.62%)	57.11 (0.05)	
*Vaccine combination*					
Monovalent	9	2989	94.04% (87.80–98.20%)	97.12% (< 0.01)	< 0.001
Hexavalent	2	283	95.55% (92.71–97.76%)	NC	
Pentavalent	11	2875	83.87% (78.04–88.97%)	93.18% (< 0.01)	
Heptavalent	1	117	88.03% (80.91–92.74%)	NA	
Tetravalent	1	279	71.68% (66.13–76.65%)	NA	
*Vaccine type*					
Recombinant	20	6422	89.06% (84.29–93.09%)	96.69% (< 0.01)	0.405
Plasma-derived	5	1606	91.92% (87.26–95.65%)	83.62% (< 0.01)	

*NC*  not computed (*I*^2^ and *p* value not computed because of insufficient data); *NA* not applicable

### Meta‐regression analysis

The meta-regression model showed no statistically significant association between the sample size and seroprotection rate (*p* = 0.703) (Additional file 3: Fig. S2). Similarly, the association between the publication year and seroprotection rate was not statistically significant (*p* = 0.368) (Additional file 3: Fig. S2). Both the sample size and publication year did not account for any percentage in the between-study variance. Furthermore, the meta-regression showed that approximately 0.81%, 27.75%, 6.68%, 17.84%, and 0% of the between-study variance was explained by region, assay method, vaccine dose, vaccine combination, and vaccine type respectively.

### Leave-one-out analysis

The seroprotection rate did not markedly change with the omission of each study in turn, indicating no strong influential studies. When compared with other studies, the omission of Nlend, 2016 (90.24% 95%CI   86.97–93.09%) and Madhi, 2011a (88.53%; 95% = 84.99–91.65%) had a relatively larger positive and negative influence on the pooled rate, respectively.

### Publication bias

**Fig. 4:**
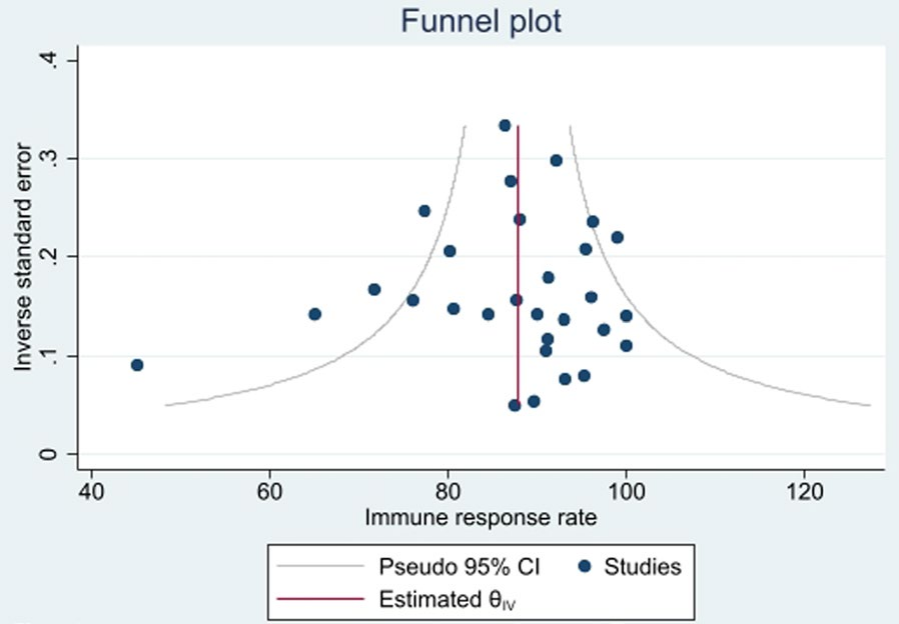
Funnel plot of included studies

The funnel plot of the studies included in the review suggests no publication bias (Fig. 4). The absence of publication bias was further confirmed by Egger’s test (*p* = 0.727).

### Factors associated with seroprotection after HBV vaccination

**Fig. 5:**
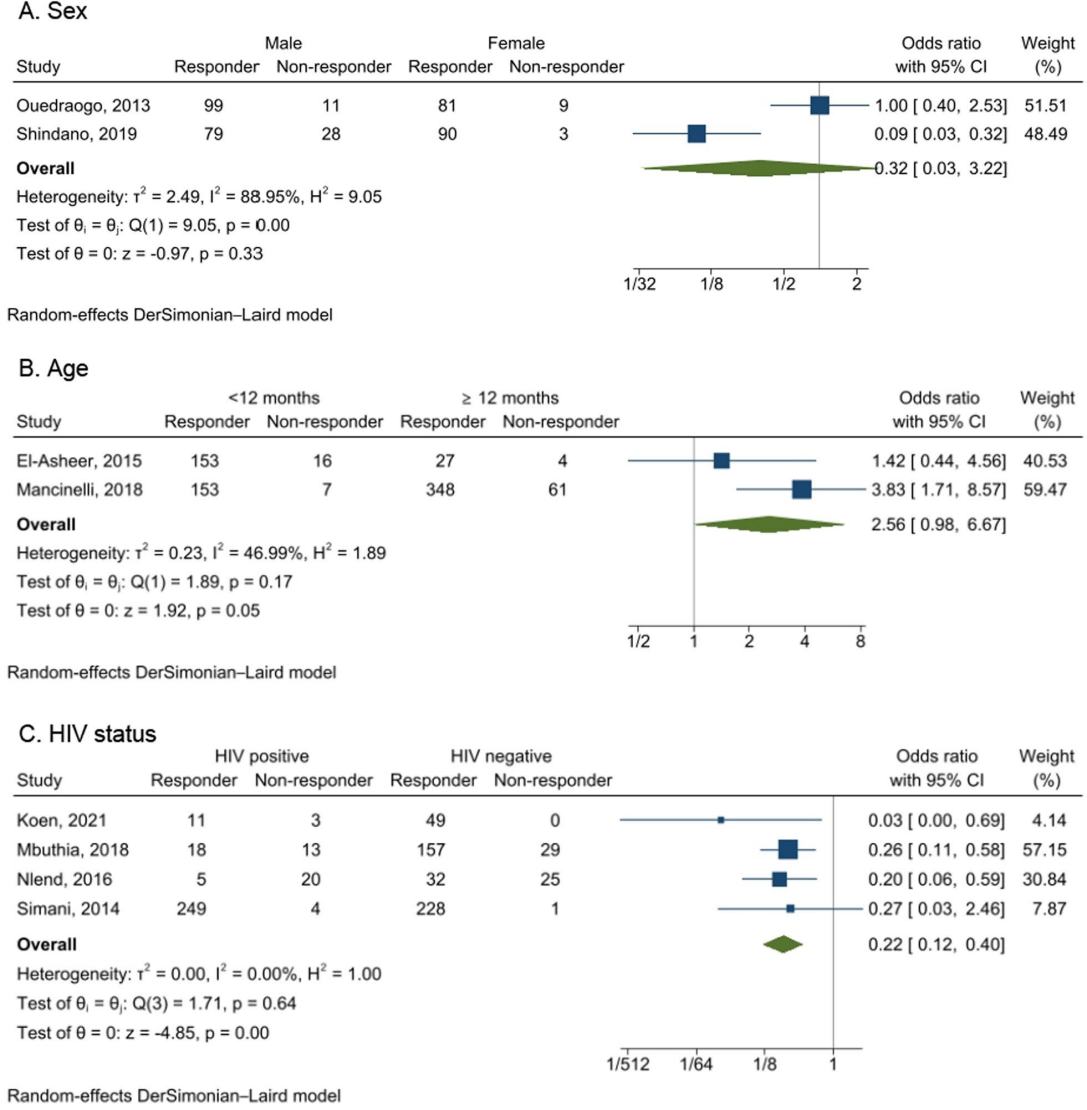
Forest plots of the factors associated with seroprotection after HBV vaccination among children under 5 years in Africa

Sex, age, and HIV status were the only factors reported by at least two studies for under-five children. There was no significant difference in the seroprotection rate between males and females (OR = 0.32, 95%CI   0.03–3.22) (Fig. 5A). Similarly, the seroprotection rate was not significantly different between children less than 12 months and children ≥ 12 months (OR = 2.56, 95%CI   0.98–6.67) (Fig. 5B). However, HIV-positive children had lower odds of achieving seroprotection when compared with HIV-negative children (OR = 0.22, 95%CI   0.12–0.40) (Fig. 5C).

## Discussion

In this study, we estimated the seroprotection rate among children under 5 years who completed three or four doses of HBV vaccine in Africa. Our results indicated that about 89% of children achieved seroprotection after HBV vaccination. This finding indicates a high immunogenicity of HBV vaccine among children and further supports the recommendation for country implementation for HBV infection prevention and control [18].

While we did not find similar studies at the regional level for comparison, the seroprotection rate among children in this study is consistent with a similar study in Iran which reported a pooled rate of 89% (95% CI   86–93%) among children under 5 years of age [81]. However, our result was higher than the rates reported by Le and colleagues in the US [82]. In their cross-sectional study of data for children and adolescents from the National Health and Nutrition Examination Survey (NHANES) from 1999 to 2016, among children aged 2–5 years who completed the HBV vaccine series, the seroprotection rate increased from 60.7% (95% CI   48.8–71.4%) to 65.2% (95% CI   57.4–72.3%) (protective immune response was defined as anti-HBs titer > 10 IU/L from 1999 to 2006 and ≥ 12 IU/L from 2007 to 2016). A small study in rural areas in Yemen also found a lower rate, with 72.2% of the under-five children having an anti-HBs level ≥ 10 IU/L [83]. The reasons for these differences are not apparent. The nature of the vaccines or vaccination may be responsible. Further investigation into the possibility of geographical variations in immune response to HBV vaccine is warranted.

Stratified by different groups, the results showed variations in the seroprotection rates. For instance, there was a significant variation in the assay method with the chemiluminescence assay having the highest immune response rate at 97.61% (95%CI   94.30–99.60%). Although the sensitivity and specificity of test assays vary by manufacturer, automated assays such as chemiluminescence tend to detect higher values of anti-HBs [84]. Compared with enzyme immunoassay which is more commonly used, chemiluminescence immunoassay has a lower turnaround time and requires less technical expertise [85]. There was also a significant subgroup difference by vaccine combination. Previous studies comparing the immunogenicity between hexavalent and pentavalent [86] and monovalent and pentavalent [87] vaccines found similar rates. Thus, more evidence is needed on this possible variation by vaccine combination. Of note, we did not find any significant difference between plasma-derived vaccine and recombinant vaccine. Safety concerns associated with plasma-derived HBV vaccine relegated its use [17]. Interestingly, the pooled seroprotection rate with four doses (i.e., a birth dose and three doses) was significantly higher than three doses, demonstrating the importance of HepB-BD. Despite the WHO recommendations of universal HepB-BD and its cost-effectiveness [88], many countries in Africa have not introduced routine HepB-BD into their national immunization programs due to reasons such as cost of implementation, the high proportion of non-institutional delivery, and limited evidence on the burden of HBV and perinatal transmission [19, 89–91].

From the few studies included in this meta-analysis, HIV was associated with lower odds of achieving seroprotection. Although there is no comparative study among children,  our finding is consistent with results observed among adults in Africa [92]. The lack of protective immune response among people living with HIV has been linked with reduced CD4 cell count and B-cell dysfunction [35, 38] and factors such as viral load, sex, and age also influence immune response to HBV vaccine among people living with HIV [92, 93]. Our finding further supports routine PVST for children living with HIV after completion of the primary series. Although not included in our meta-analysis, other factors such as vitamin A supplementation [70] and EBV infection [58] were reported to be associated with immune response by single studies. These factors need to be further explored in future studies.

Several factors including perinatal host, nutritional, environmental, and immunization-related factors such as suboptimal dosing, site of administration, and reduced potency due to poor vaccine storage and handling conditions, could have accounted for the nonresponse among the under 5 children in the studies [31, 94]. While the cellular mechanisms involved in nonresponse to HBV vaccination remain unclear, impaired lymphocyte activation has been implicated [38, 95]. Interventions to address nonresponse among children will depend on largely the prevailing health status. A booster HBV vaccine dose can induce anamnestic response in most children without an immune response [96]. However, it is not recommended by the WHO for persons with normal immune status who have received a full primary course of HBV vaccine [18]. Some studies indicate long-term protection of the HBV vaccine regardless of the level of measurable anti-HBs titer [97–99]. On the other hand, for non-responding HIV-positive children, a second HBV vaccine series using larger or additional doses is recommended [34, 100, 101] or vaccination could be repeated after an increase in CD4 cell count or viral load suppression [18]. New vaccines with simplified schedules, and that can elicit higher anti-HBs response more rapidly are currently available and underway [17, 102]. However, these vaccines are still mostly for adults.

This systematic review has a few limitations. We only searched three databases and did not conduct hand-searches. The cutoff for seroprotection was not consistent across the study. Although most of the studies used ≥ 10 IU/L, four studies [58, 59, 66, 67] used > 10 IU/L. Our subgroup analysis showed no statistically significant difference between the two (data not shown). Secondly, the interval between the last dose and the immunoassay was not reported in some studies and varied across those that reported it. The optimal timing of PVST is within one to two months of the final dose. An immune response may decrease with longer intervals [103, 104]. In two studies, [62, 65] some of the participants received investigational vaccines that were yet to be licensed for use in the general public as at the time of the study. We were only able to examine a few factors associated with seroprotection because they were reported by a small number of the included studies for children under 5 years. Some of the pooled rates were also from a limited number of studies, thus the results should be interpreted cautiously.

## Conclusions

Our study findings indicate that HBV vaccine induces a protective immune response in the majority of children under 5 years who complete three or four doses. However, the rate is lower among children living with HIV. This calls for interventions to timely identify and address nonresponse to HBV vaccine, particularly among children living with HIV. The high immunogenicity of HBV vaccine observed in this study further supports the need to scale up HBV vaccination coverage rates among under-five children in Africa. More studies are needed to better understand the factors associated with immune response to HBV vaccine among children under 5 years in Africa.

### Supplementary Information

**Additional file 1.** Search Strategy for PubMed.

**Additional file 2: Fig. S1.** Forest plots of the seroprotection rates after HBV vaccination among children under 5 years in Africa by (**A**) study region, (**B**) vaccine dose, (**C**) assay method, (**D**) vaccine combination, and (**E**) vaccine type.

**Additional file 3: Fig. S2.** Meta-regression of the seroprotection rates after HBV vaccination children under 5 years in Africa against (**A**) sample size and (**B**) publication year.

## Data Availability

The data set used in the meta-analysis is available from the corresponding author on request.
