# Supplementary material for: Immune response to hepatitis B vaccine among children under 5 years in Africa: a meta-analysis
Source: Trop Med Health. 2024 Apr 1;52:28. doi: 10.1186/s41182-024-00594-4 (PMC10983738; doi:10.1186/s41182-024-00594-4)
Supplement: Supplementary file 1 — Additional file 1. Search Strategy for PubMed. [file 41182_2024_594_MOESM1_ESM.docx]

Additional File 1

Search Strategy for PubMed

“hepatitis B” OR HBV OR HepB

AND

vaccin* OR immuni*

AND

“post vaccine*” OR post-vaccin* OR PVST OR “immune response” OR immunogeni* OR seroprotect* OR sero-protect* OR protect* OR response OR efficacy OR effective* OR effect

AND

Infan* OR Child* OR Newborn* OR Baby OR Neonate* OR Pediatric* OR Peadiatric*

AND

Africa OR “sub-Saharan Africa” OR Algeria OR Angola OR Benin OR Botswana OR “Burkina Faso” OR Burundi OR Cameroon OR “Cape Verde” OR “Central African Republic” OR Chad OR Comoros OR “Democratic Republic of the Congo” OR DRC OR Congo OR “Republic of the Congo” OR Djibouti OR Egypt OR “Equatorial Guinea” OR Eritrea OR Ethiopia OR Gabon OR Gambia OR Ghana OR Guinea OR Guinea-Bissau OR “Ivory Coast” OR “Cote d’Ivoire” OR Kenya OR Lesotho OR Liberia OR Libya OR Madagascar OR Malawi OR Mali OR Mauritania OR Mauritius OR Morocco OR Mozambique OR Namibia OR Niger OR Nigeria OR Rwanda OR “Sao Tome” OR Principe OR Senegal OR Seychelles OR “Sierra Leone” OR Somalia OR “South Africa” OR “South Sudan” OR Sudan OR Swaziland OR Tanzania OR Togo OR Tunisia OR Uganda OR Zambia OR Zimbabwe
