# Supplementary material for: Immune response to hepatitis B vaccine among children under 5 years in Africa: a meta-analysis
Source: Trop Med Health. 2024 Apr 1;52:28. doi: 10.1186/s41182-024-00594-4 (PMC10983738; doi:10.1186/s41182-024-00594-4)

Supplementary Figure 1. Forest plots of the seroprotection rates after HBV vaccination among children under five years in Africa by (A) study region, (B) vaccine dose, (C) assay method, (D) vaccine combination, and (E) vaccine type

1. Study region


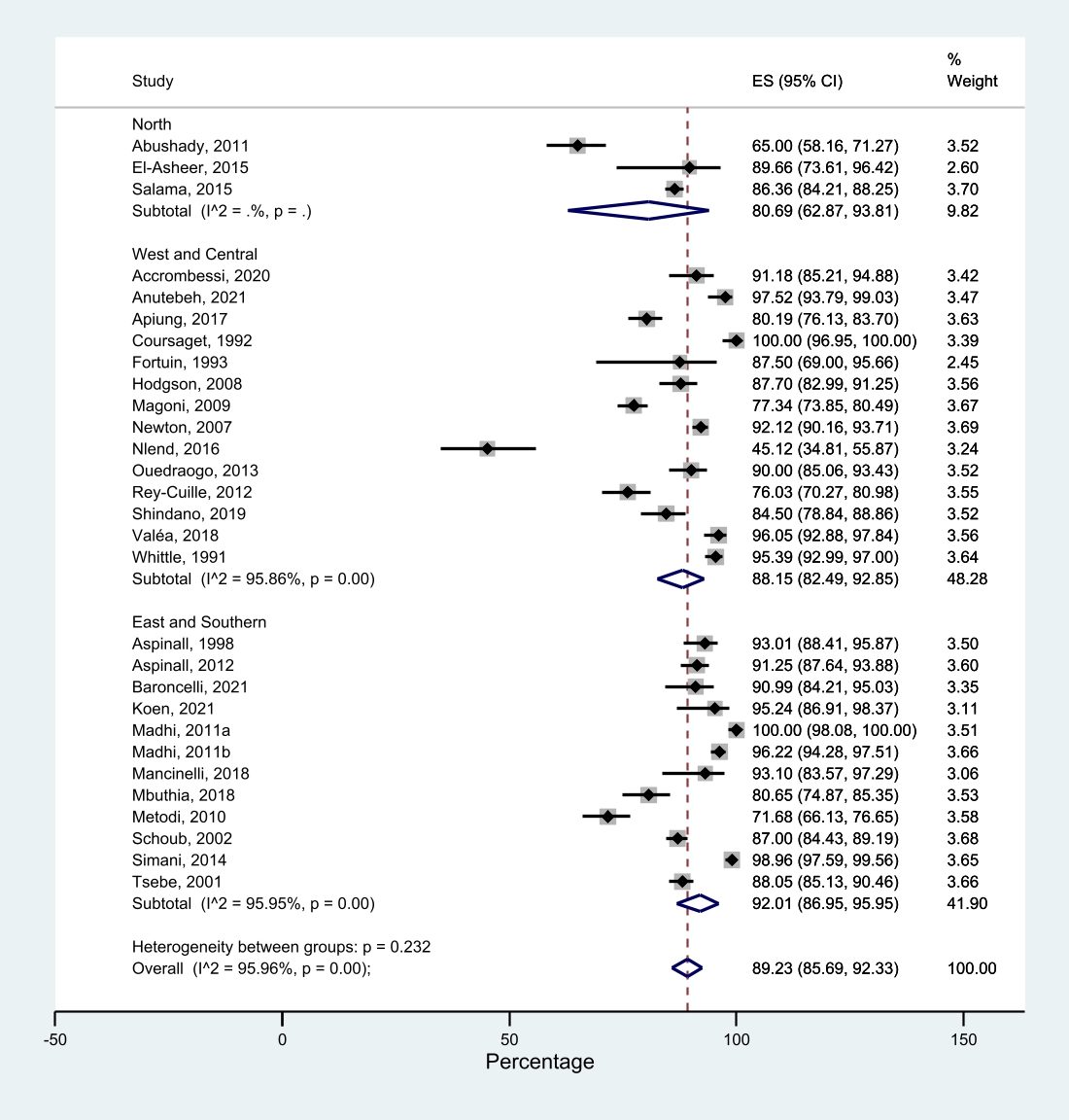


1. Vaccine dose


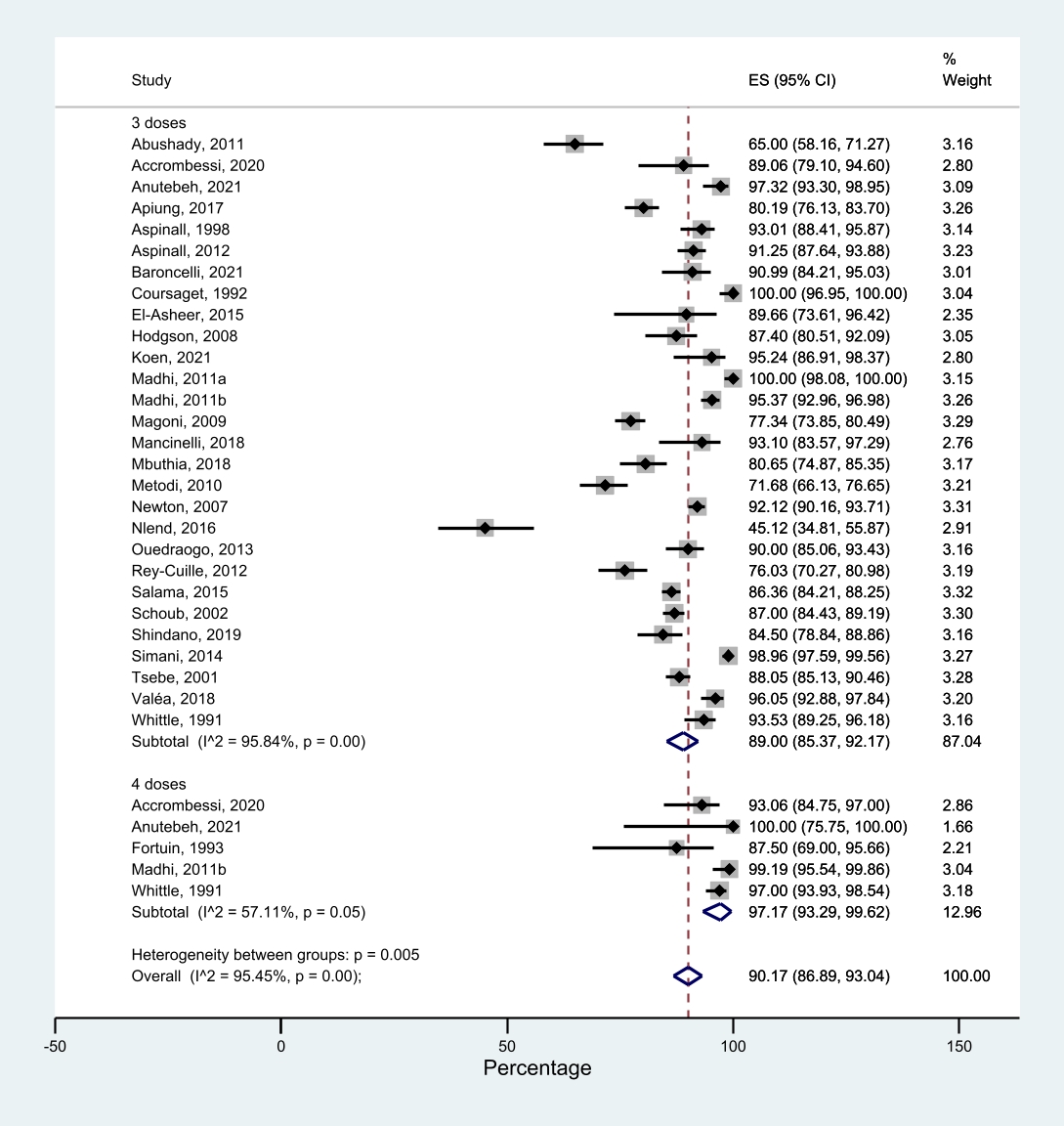


1. Assay method


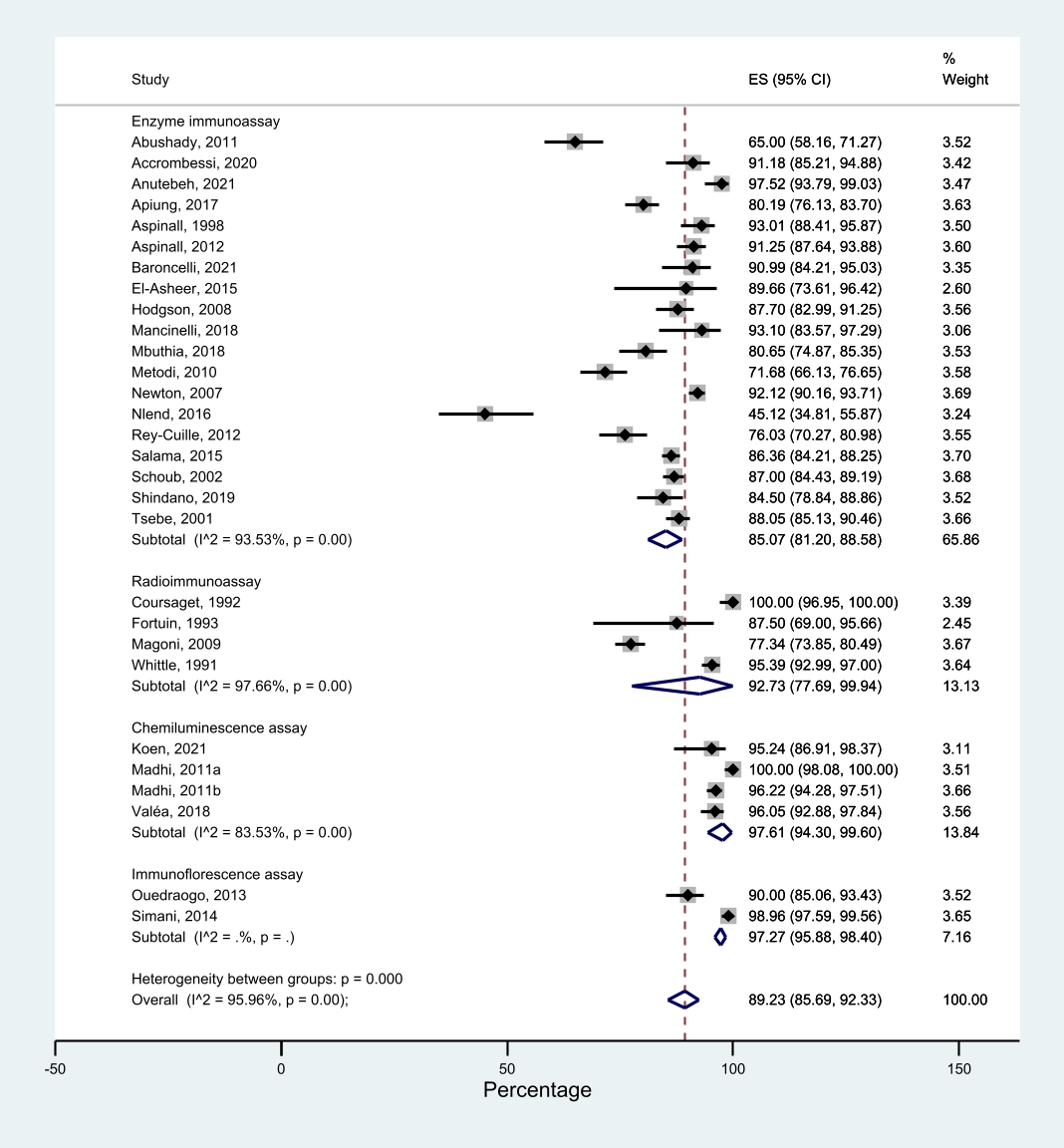


1. Vaccine combination


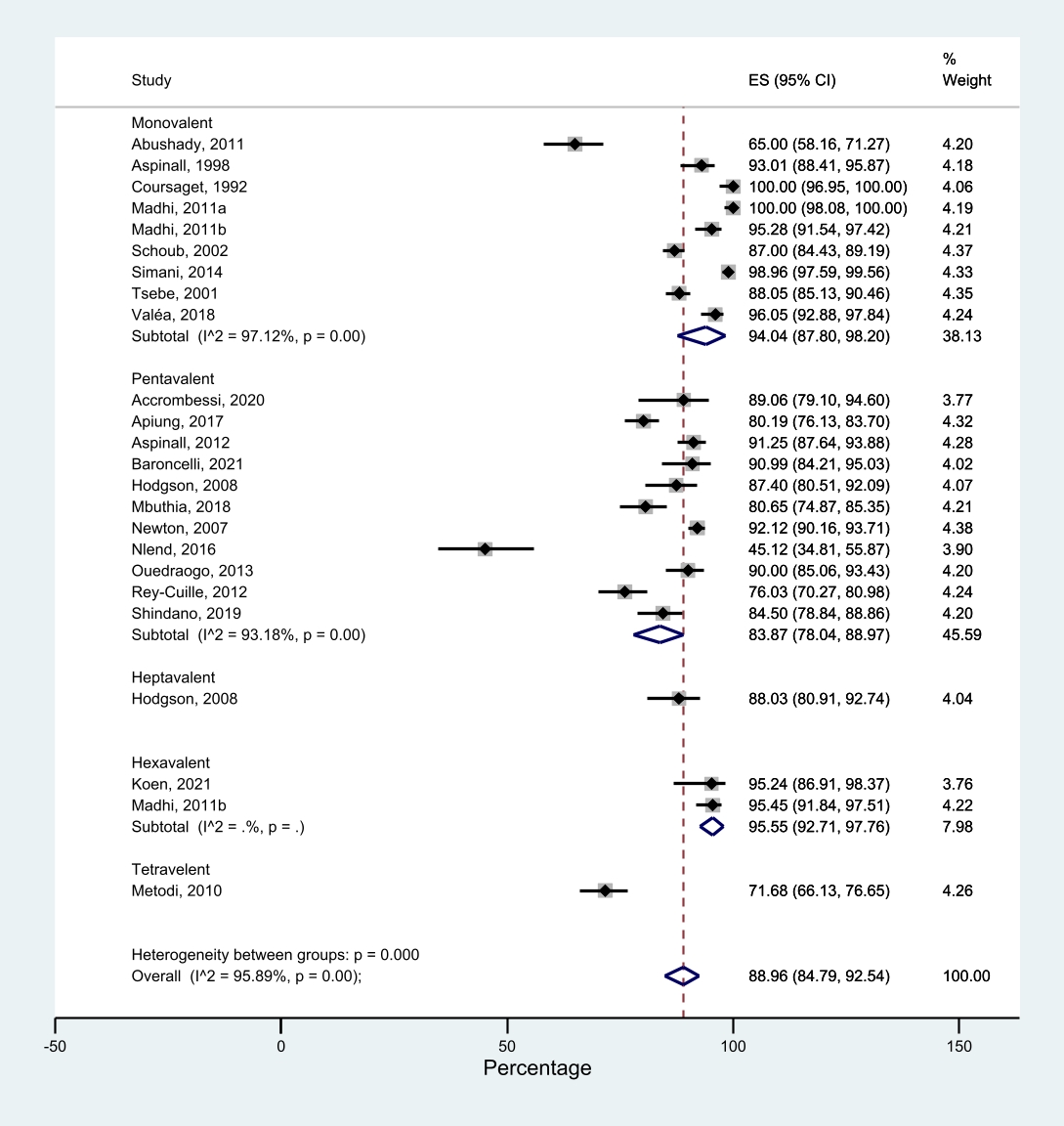


1. Vaccine type


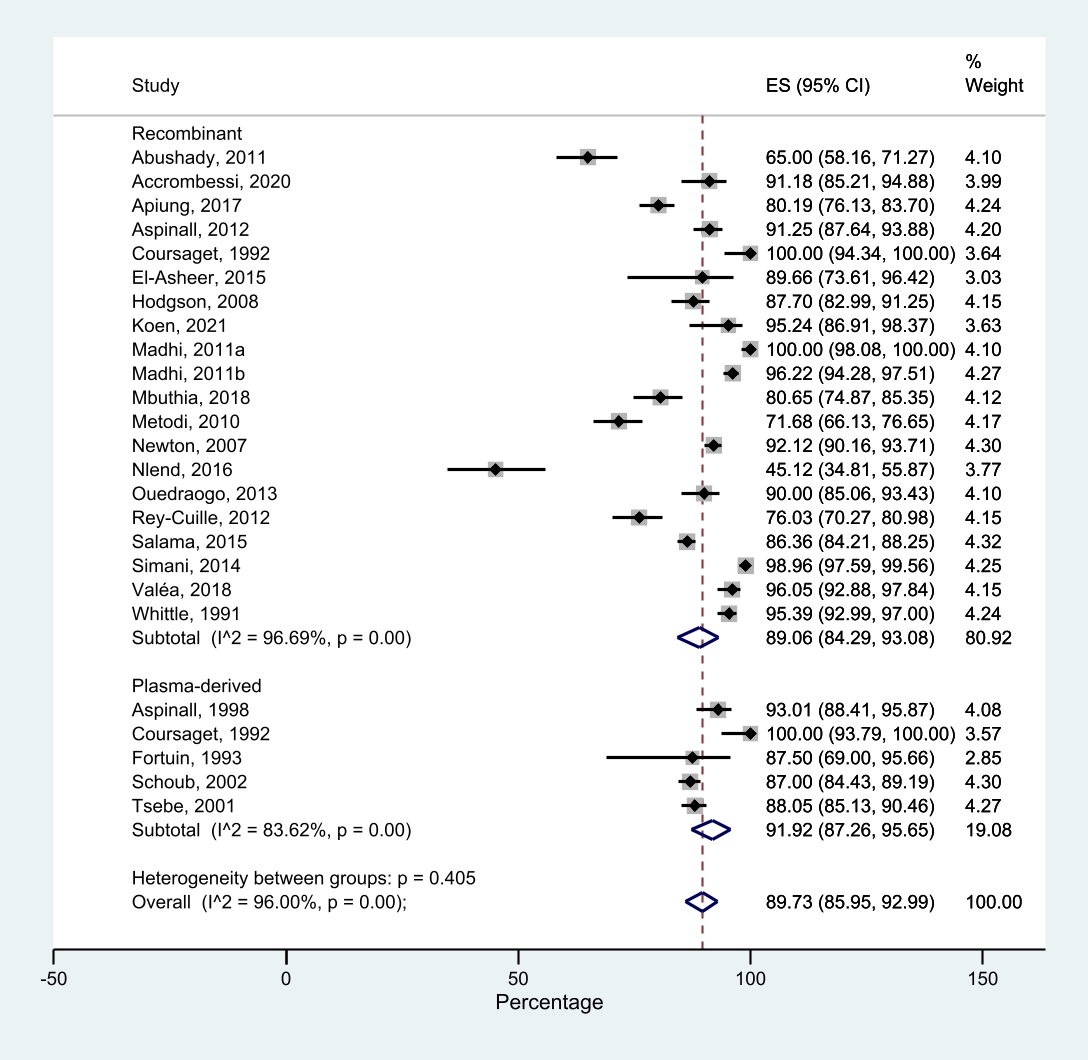

Supplement: Supplementary file 2 — Additional file 2: Fig. S1. Forest plots of the seroprotection rates after HBV vaccination among children under 5 years in Africa by (A) study region, (B) vaccine dose, (C) assay method, (D) vaccine combination, and (E) vaccine type. [file 41182_2024_594_MOESM2_ESM.docx]
