# Supplementary material for: Immune response to hepatitis B vaccine among children under 5 years in Africa: a meta-analysis
Source: Trop Med Health. 2024 Apr 1;52:28. doi: 10.1186/s41182-024-00594-4 (PMC10983738; doi:10.1186/s41182-024-00594-4)

Supplementary Figure 2. Meta-regression of the seroprotection rates after HBV vaccination among children under 5 years in Africa against (A) sample size and (B) publication year

1. Sample size


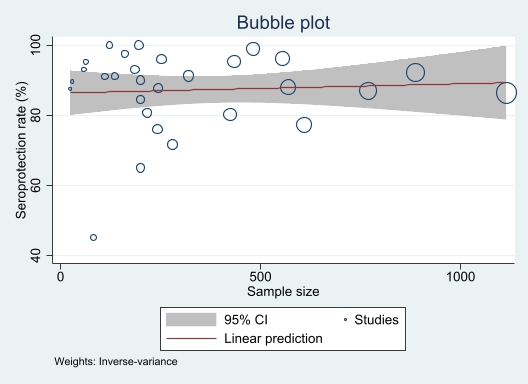


1. Publication year


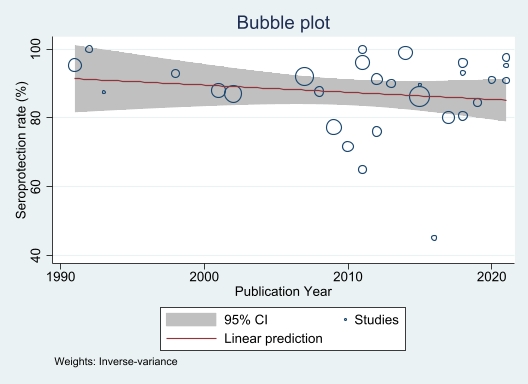

Supplement: Supplementary file 3 — Additional file 3: Fig. S2. Meta-regression of the seroprotection rates after HBV vaccination children under 5 years in Africa against (A) sample size and (B) publication year. [file 41182_2024_594_MOESM3_ESM.docx]
